# Recurrent spindle cell carcinoma of the lung successfully treated by chemoimmunotherapy

**DOI:** 10.1002/rcr2.757

**Published:** 2021-05-07

**Authors:** Tomohiro Akaba, Yuno Shiota, Fumi Onizawa, Tamami Isaka, Yoji Nagashima, Etsuko Tagaya

**Affiliations:** ^1^ Department of Respiratory Medicine Tokyo Women's Medical University Tokyo Japan; ^2^ Department of Thoracic Surgery Tokyo Women's Medical University Tokyo Japan; ^3^ Department of Surgical Pathology Tokyo Women's Medical University Tokyo Japan

**Keywords:** Chemoimmunotherapy, sarcomatoid carcinoma, spindle cell carcinoma

## Abstract

There is limited evidence for the treatment of sarcomatoid lung carcinoma, especially spindle cell carcinoma (SpCC) because of the rarity of disease. Although the efficacy of combination of chemotherapy and immunotherapy (i.e. chemoimmunotherapy) for non‐small cell lung cancer (NSCLC) is well recognized, the effect for SpCC is not fully elucidated. A 72‐year‐old woman underwent surgical resection for the treatment of stage IIIA SpCC. Recurrence occurred three months after surgery, and then she received combination of carboplatin, paclitaxel, bevacizumab, and atezolizumab. A clinically favourable response was achieved by four cycles of chemoimmunotherapy and sequential maintenance therapy with bevacizumab and atezolizumab. The prognosis of advanced SpCC is generally poor, but chemoimmunotherapy can be a good therapeutic option for the first‐line treatment of SpCC.

## Graphical abstract

Here, we report a case of spindle cell carcinoma (SpCC), which recurred after surgical resection and showed a good response to combination of carboplatin, paclitaxel, bevacizumab, and atezolizumab as a first‐line treatment.

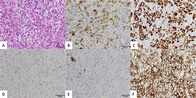

## Introduction

Spindle cell carcinoma (SpCC) is a rare subgroup of the sarcomatoid carcinoma (SC) [1]. The prognosis of SpCC is poor despite the very best of treatment. Recently, the effectiveness of immune checkpoint inhibitor was proven in patients with SC [2], but to date, the efficacy of combination of cytotoxic chemotherapy and immune checkpoint inhibitor (i.e. chemoimmunotherapy) was not well studied. Here, we report a case of SpCC, which recurred after surgical resection and showed a good response to combination of carboplatin, paclitaxel, bevacizumab, and atezolizumab as a first‐line treatment.

## Case Report

**Figure 1:**
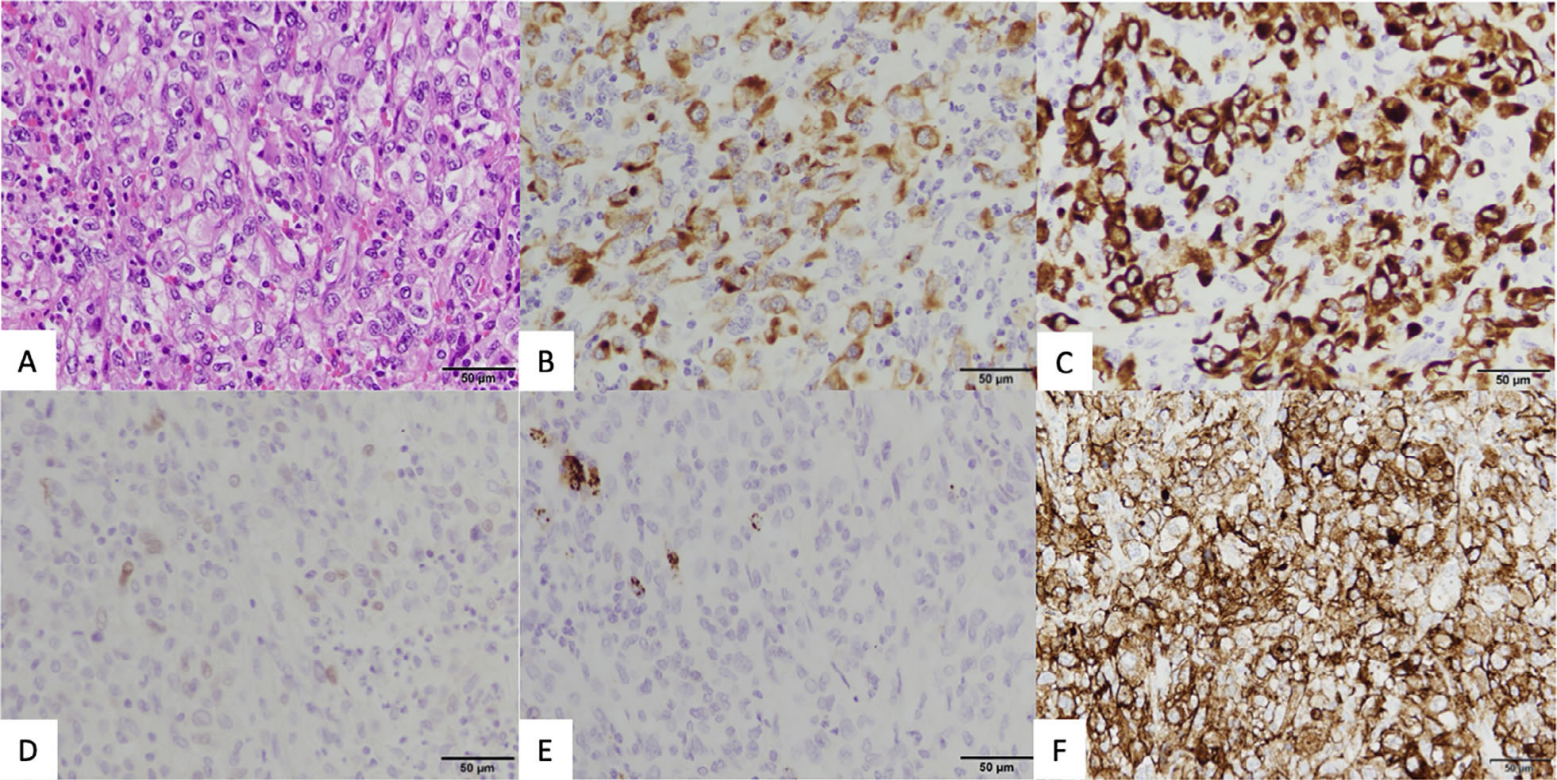
Pathological findings of resected right upper lung lobe. (A) Accumulation of spindle‐shaped cells was seen. (B) Tumour cells were positive for immunohistochemistry of cytokeratin, (C) clone AE1/AE3, and (D) clone CAM5.2. (D) Thyroid transcription factor‐1 (TTF‐1) was weakly positive, (E) while napsin A was negative. (F) Programmed death‐ligand 1 (PD‐L1)‐positive cells accounted for 100%.

**Figure 2:**
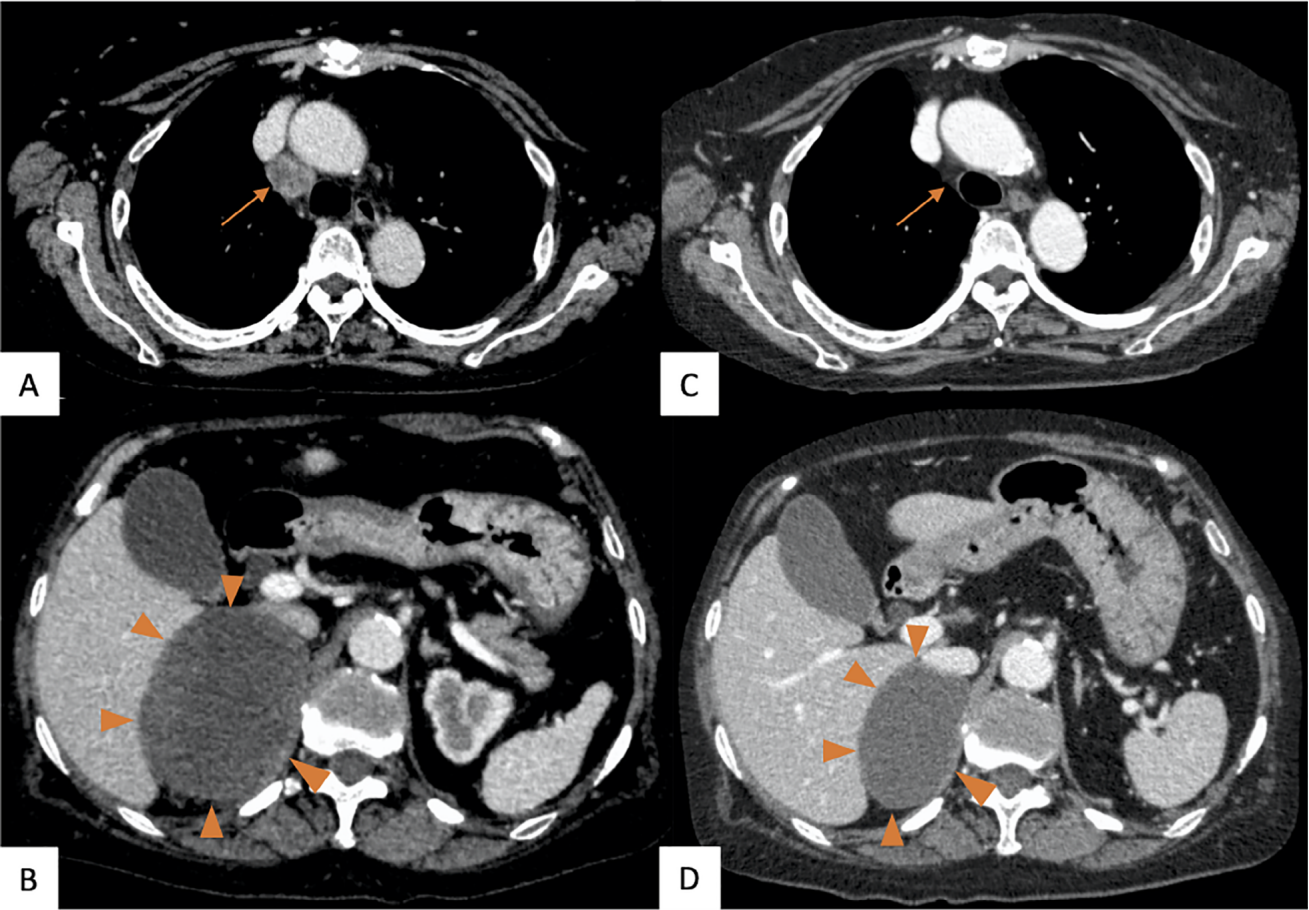
Computed tomography (CT) scans before and after chemoimmunotherapy. (A, B) Metastases to the mediastinal lymph node (arrow) and right adrenal (arrowheads) were observed three months after the surgery. (C) The shrinkage of the mediastinal lymph node (arrow) was confirmed after four courses of chemoimmunotherapy. (D) The right adrenal gland reduced in size from 81 × 65 to 61 × 43 mm (arrowheads).

A 72‐year‐old woman with a 10‐pack‐year smoking history was referred to our department due to a lung mass in right upper lobe. She has been treated with rheumatoid arthritis and received oral prednisolone with tocilizumab. Slight increase of pro‐gastrin‐releasing peptide (ProGRP) was seen (147.8 pg/mL), but other tumour markers including carcinoembryonic antigen (CEA) and cytokeratin 19 fragment (CYFRA) were within normal limit. The cytology obtained by bronchoscopy showed class V, while histological type was uncertain. After whole body check‐up, clinical stage of her lung cancer was classified as stage IIIA (cT2aN2M0). She underwent surgical resection of the right upper lobe with lymph node dissection. The resected lung showed accumulation of spindle‐shaped cells, suggesting the diagnosis of SpCC (Fig. 1A–E). No metastasis was found in the hilar and mediastinal lymph nodes simultaneously resected. Her pathological stage after surgery was stage IIB (pT3N0M0). Although gene mutation for *EGFR*, *ALK*, and *ROS1* was absent, high programmed death‐ligand 1 (PD‐L1) expression was detected (100% positive using immunohistochemistry (clone 22C3; Dako PharmDx, United States)) (Fig. 1F). She did not receive an adjuvant chemotherapy because her rheumatoid arthritis worsened after the discontinuation of tocilizumab and lack of evidence for adjuvant chemotherapy for SpCC. Three months after the surgery, the contrast computed tomography (CT) showed the enlargement of the mediastinal lymph node and right adrenal gland, which suggested the metastatic recurrence of disease (Fig. 2A, B). Then, she received combination of carboplatin, paclitaxel, bevacizumab, and atezolizumab. After four cycles of treatment, both the swelling mediastinal lymph node and right adrenal metastasis shrank in size, confirming the Response Evaluation Criteria in Solid Tumors Criteria partial response (Fig. 2C, D). No serious immune‐related adverse event was observed. At present, she has been receiving bevacizumab and atezolizumab as a maintenance therapy for 15 months.

## Discussion

In this study, we showed a very rare type of lung cancer well responded to chemoimmunotherapy. According to the World Health Organization (WHO) classification, SpCC is a group of SC, which is defined as a group of poorly differentiated non‐small cell lung cancer (NSCLC) with components of sarcoma or sarcoma‐like (spindle cell or giant cell) differentiation [1]. SC accounts for approximately 1% of all lung cancers and most of the SCs are pleomorphic carcinomas; SpCC accounts for less than 10% of SC.

Because of the rarity of disease, there is no standard strategy for the treatment of SC including SpCC. Surgical resection should be considered if disease stage is early, but the recurrence of SC is frequently seen [1]. Although previous case reports showed the efficacy of carboplatin, paclitaxel, and bevacizumab regimen or germanium sesquioxide, SC patients are usually chemotherapy resistant. The prognosis of advanced SC is considered very poor despite the conventional chemotherapy for NSCLC and median survival duration is only 6.3 months [3].

According to the recently published report, immune checkpoint inhibitor as second‐line or beyond significantly prolonged overall survival among the patients with SC [2]. In that study, the median PD‐L1 expression was 70%, which was consistent with the previous report showing that PD‐L1 expression in SC tended to be high [4]. There was a trend towards that higher PD‐L1 expression and tumour mutational burden (TMB) showed good response to immune checkpoint inhibitor. Although we did not evaluate TMB, high PD‐L1 expression may be a positive predictor of treatment efficacy in this case. Socinski et al. evaluated the effect of carboplatin, paclitaxel, bevacizumab, and atezolizumab regimen as a first‐line therapy for the treatment of advanced non‐squamous NSCLC [5]. This multicentre randomized trial showed that the addition of atezolizumab significantly improved the outcome regardless of PD‐L1 expression compared with chemotherapy alone. Although further studies are required to confirm the efficacy of this regimen for SpCC patients, as the current case showed, it has the possibility that the combination of immune checkpoint inhibitor and cytotoxic chemotherapy synergistically enhance the effect and improve the treatment outcome.

In conclusion, combination of carboplatin, paclitaxel, bevacizumab, and atezolizumab can be a good treatment option as a first‐line therapy for patients with SpCC. Clinical trial to evaluate the efficacy of this regimen in SC, especially SpCC patients, is warranted.

### Disclosure Statement

Appropriate written informed consent was obtained for publication of this case report and accompanying images.

### Author Contribution Statement

Tomohiro Akaba: drafting the draft. Yuno Shiota: supervision, revising the draft. Fumi Onizawa: revising the draft. Tamami Isaka: revising the draft, Yoji Nagashima: revising the draft. Etsuko Tagaya: supervision and final approval of the draft.

## References

[1] Park JS , Lee Y , Han J , et al. 2011. Clinicopathologic outcomes of curative resection for sarcomatoid carcinoma of the lung. Oncology 81(3–4):206–213. 22076573 10.1159/000333095

[2] Domblides C , Leroy K , Monnet I , et al. 2020. Efficacy of immune checkpoint inhibitors in lung sarcomatoid carcinoma. J. Thorac. Oncol. 15(5):860–866. 31991225 10.1016/j.jtho.2020.01.014

[3] Vieira T , Girard N , Ung M , et al. 2013. Efficacy of first‐line chemotherapy in patients with advanced lung sarcomatoid carcinoma. J. Thorac. Oncol. 8(12):1574–1577. 24389441 10.1097/01.JTO.0000437008.00554.90

[4] Vieira T , Antoine M , Hamard C , et al. 2016. Sarcomatoid lung carcinomas show high levels of programmed death ligand‐1 (PD‐L1) and strong immune‐cell infiltration by TCD3 cells and macrophages. Lung Cancer 98:51–58. 27393506 10.1016/j.lungcan.2016.05.013

[5] Socinski MA , Jotte RM , Cappuzzo F , et al. 2018. Atezolizumab for first‐line treatment of metastatic nonsquamous NSCLC. N. Engl. J. Med. 378(24):2288–2301. 29863955 10.1056/NEJMoa1716948

